# Temporal changes in bird communities in areas with different histories of fire disturbance in highland grasslands of Brazil

**DOI:** 10.1371/journal.pone.0243070

**Published:** 2020-12-02

**Authors:** Eduardo Chiarani, Maurício Bettio, Carla Suertegaray Fontana

**Affiliations:** Laboratório de Ornitologia, Museu de Ciências e Tecnologia, Programa de Pós-Graduação em Ecologia e Evolução da Biodiversidade, Pontifícia Universidade Católica do Rio Grande do Sul—PUCRS, Porto Alegre, Rio Grande do Sul, Brazil; University of Brasilia, BRAZIL

## Abstract

Despite the importance and ubiquity of grasslands, the degradation and the loss of these habitats have negatively affected bird populations throughout the world. The use of fire to manage grassland areas in some regions of southern Brazil can help to maintain these areas but can also influence the bird community in different ways. We assessed temporal changes in richness, abundance, and composition of bird communities in areas with different histories of fire disturbance in highland grasslands of southern Brazil, the most extensive remnant of grassland of the Atlantic Forest domain. We censused birds during four breeding seasons (2015–2018), through point counts in areas burned only once in the last ten years (OF, n = 3), areas burned annually (AF, n = 2), and areas without fire in the last ten years (WF, n = 2). In OF the richness, abundance, and species composition changed in the year of the fire, compared to the previous year, and returned to the initial values two years later. In AF and WF we found some differences among the years, but not with an equally clear pattern. Three of the six grassland associated species assessed individually for density responded significantly to temporal habitat modification since the fire. Our results show that two years without fire were enough time for the bird community to recover after a fire, but some responses are species-specific. Therefore, fire can be used as a management tool for grasslands and may help in the conservation of birds of southern Brazil, as long as with a minimum interval between fires in an area is guaranteed.

## Introduction

Grasslands occupy about 13.7 million ha of South Brazil and have been undergoing an extensive transformation in recent decades, mainly from the conversion of native grassland areas to agriculture or afforestation [1, 2]. The degradation and loss of grassland areas have negatively affected bird populations throughout the world [1]. The biodiversity in southern Brazilian highland grasslands, located in northeastern Rio Grande do Sul and the states of Santa Catarina and Paraná, has also been impacted by anthropic actions [3–5]. This region houses about 70% of the bird species associated with grassland landscapes in southeastern South America, including endemic, migratory and/or threatened species [1, 6–8].

The two main ways of managing grasslands are fire and grazing, and these disturbances are usually associated with cattle raising, an activity that allows combining production and conservation in southern Brazil [3, 9, 10]. In southern Brazilian highland grasslands, fire is traditionally used to manage them at the end of winter, to burn the accumulated biomass and stimulate the regrowth of vegetation for cattle feed [11, 12]. In the last years, laws have been introduced to allow the use of controlled fire in grasslands in some municipalities of northeastern Rio Grande do Sul state, but this decision is usually based more on political and cultural issues than on scientific studies that assess the impacts of fire on animal and plant communities. Although the use of fire is already recommended as a management tool for protected areas in Brazil, its application is not yet a reality in these areas, and this issue is still a taboo [13, 14].

It is known that in the absence of any disturbance (e.g., grazing or fire), grasslands show a high dominance of a few species of caespitose grasses (that form tussocks) and a low diversity of forbs, resulting in a homogenization of the vegetation structure [2, 15] and a consequent reduction of the bird diversity [16, 17]. In the long term, in abandoned grasslands (i.e. long periods without grazing or fire), the floristic richness can be reduced and the grassland vegetation itself can be lost due to encroachment of shrubs [12, 18, 19]. A recent study in the highlands of southern Brazil estimated in 30 years the time needed for shrubs to encroach into 99% of grasslands without management [20]. Fire exclusion also leads to the accumulation of flammable biomass and, consequently, can increase fire intensity and risk of catastrophic fire [13, 19, 21]. However, when in excess (e.g., overgrazing, cattle trampling, and high frequencies of fire) these disturbances can cause the decline of threatened bird species, mainly those dependent of tall grasslands, as observed for Rio Grande do Sul state in Brazil [5, 22]. Thus, the threshold between sustainable use and degradation seems to be subtle when we consider fire is a factor in maintaining the integrity of grasslands [9].

In North America, the probability of occurrence of several grassland bird species has decreased significantly in areas where the coverage of tall shrubs and trees has increased [23]. Some species increase in density in recently burned areas, and may be excluded from unburned areas [24–26]. For other species, the reductions in the abundance of individuals and the number of nests suggest that fire has a strong negative effect [26, 27]. Since fire can directly (nest destruction) or indirectly (changes in vegetation structure) affect the bird community in different ways [28], studies that assess the effects of burning are necessary for the proper management of grassland areas.

In South America most of the studies about fire effects on birds in non-forest habitats have been conducted in Argentina (e.g., [29–32]) or in Central Brazil (e.g., [33–36]). Few studies have specifically assessed this issue in southern Brazilian highland grasslands, and most have only compared burned and unburned areas (e.g., [37–39]). Despite the temporal scale strongly influences both the ecosystem responses to fire and the effects of fire [40], long-term studies are uncommon, and temporal aspect of the effects of certain variables (e.g., intensity, period, and frequency of fire) on bird response to fire is seldom discussed [41], particularly in Neotropical region. Therefore, approaches that evaluate temporal dynamics of fire effects, including pre- and post-fire periods, are needed to assess possible changes associated with fire in local populations and bird community. Our study is the first that considers the temporal dynamics of fire on bird community in highland grasslands of South Brazil.

Here, we assessed the parameters of the grassland bird community (richness, abundance, and composition) over four breeding seasons, in areas with different histories of fire disturbance, aiming to determine the effects of fire on grassland birds over time. We hypothesized that: (1) Bird community structure in areas with a constant history of management over time (i.e. annual fire or without fire) does not change temporally, or may present a different variation pattern when compared with areas burned occasionally; (2) Richness, abundance, and bird composition changes after an occasional fire and return to pre-disturbance levels as time goes by; and (3) Since we know that birds respond differently to variations in habitat structure caused by fire [28, 30], and differences in vegetation height is an important driver of species sorting [42], the predicted abundance of tall-grass species will reduce and the abundance of short-grass species will increase in grasslands in the year of the fire.

## Materials and methods

### Study area

**Fig 1:**
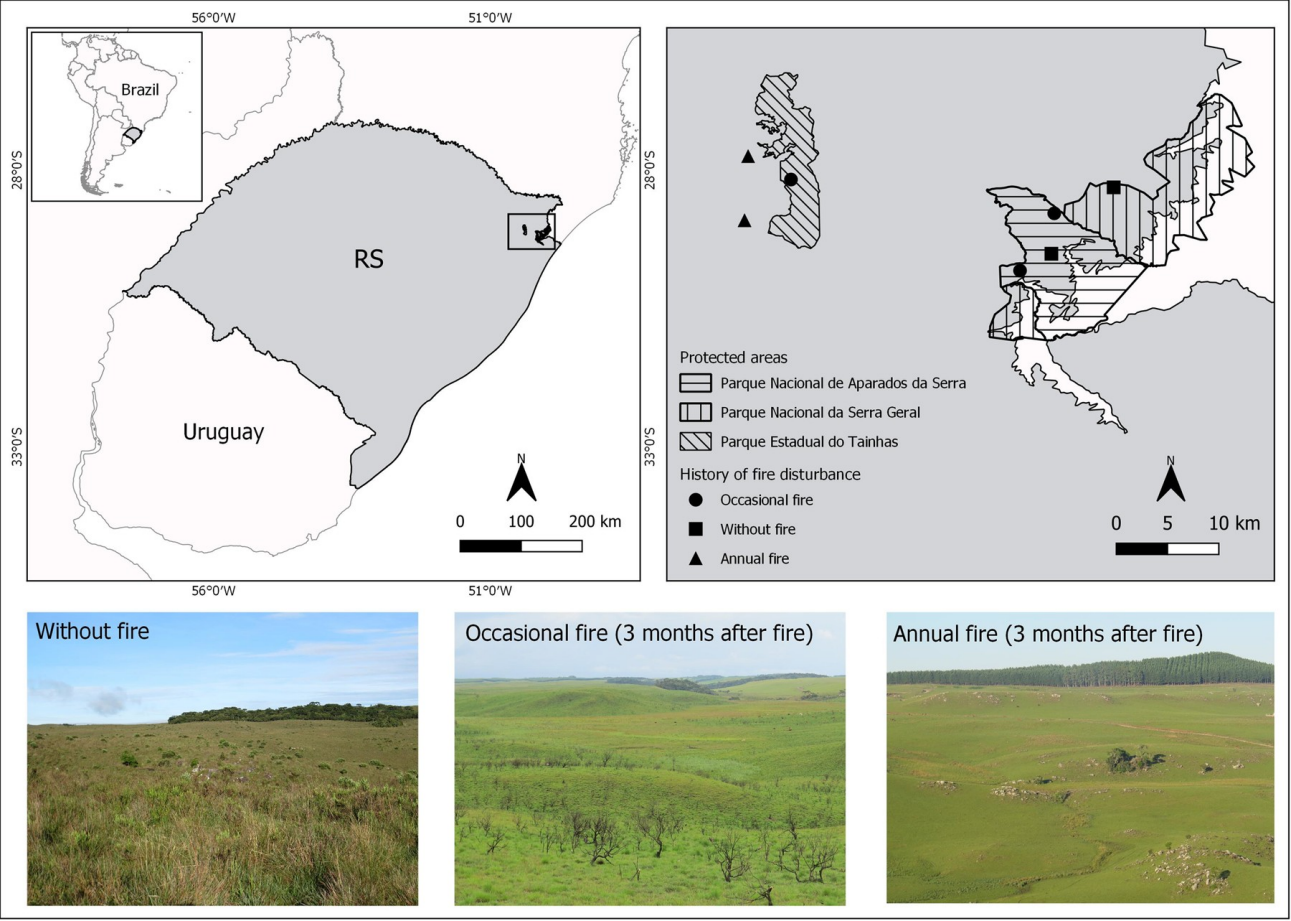
Locations of the study areas in highland grasslands in northeastern Rio Grande do Sul (RS) state, Brazil. Pictures are examples of landscapes of the three histories of fire disturbance in our study areas. Map created using QGIS software, version 3.6. Photos by EC.

The study areas are situated in the southern Brazilian highland grasslands (*sensu* [14]), in the Atlantic Forest domain. We selected seven areas of native grassland, in three protected areas (Parque Nacional de Aparados da Serra, Parque Nacional da Serra Geral, and Parque Estadual do Tainhas) and on nearby farms, in northeastern Rio Grande do Sul state (Fig 1). We categorized areas according to their histories of fire disturbance: (1) occasional fire (OF)–areas burned (accidentally) only once in the last ten years (n = 3); (2) annual fire (AF)–areas burned annually (n = 2); and (3) without fire (WF)–areas without fire in the last ten years (n = 2). The fires occurred between August and October in the study areas, even those burned accidentally. All areas (including the protected areas) are used for cattle raising and have similar low grazing pressure (0.3 to 0.5 animal unit per hectare). Our study areas are composed mainly by large extensions of native grasslands interspersed with marshes, rocky outcrops, and patches of *Araucaria* forests (*Araucaria angustifolia*). Surrounding areas also have these features and, in addition, usually have exotic pine (*Pinus* spp.) plantations and crops (mainly maize, potatoes, and soybean).

The landscape of the study areas resembles typically the grasslands in the Atlantic Forest domain of South Brazil. Southern Brazilian highland grasslands cover about 60,000 km^2^ and consist of a mosaic of grasslands and *Araucaria* forest, together with other vegetation types in a minor contribution, such as wetlands and peat bogs, with an undulating relief and mean altitude of 900–1,000 m a.s.l. [1, 5, 12, 14]. These grasslands are characterized by the dominance of perennial grass species in terms of cover and by high levels of endemism and high overall species richness, where Poaceae (mostly represented by tussock species such as *Andropogon lateralis*, *Axonopus siccus*, and *Schizachyrium tenerum*), Asteraceae, Fabaceae and Cyperaceae are the main families in species number [14, 43]. The mean annual temperature in the region ranges from 16°C to 22°C and the precipitation is evenly distributed throughout the year (1,500–2,000 mm), reaching up to 2,500 mm in certain subregions [44, 45].

### Data collection

Bird surveys were conducted during four consecutive breeding seasons (2015–2016 to 2018–2019), between November and February, the breeding period of most species in the region [46]. Birds were recorded in point counts of 10 min with an 80 m radius [47]. All individuals sighted and/or heard inside the circle were counted and their distances from the observer were estimated. Samplings were carried out from dawn to 10:00 a.m., in suitable weather conditions (without rain and with wind less than 10 km/h). The number of point counts was distributed according to the size and availability of the area in each breeding season (S1 Table), with a proportional number of points located in dry grasslands and near wetlands in each area. The minimum distance between two point-count centers was 300 m, and they were sited in open areas at a minimum distance of 150 m from the edges of other vegetation types (e.g., forests) or from fences. Each point was sampled twice per breeding season (see statistical analysis) to record the bird richness more accurately. The procedures adopted to avoid double counts and ensure independence among counts were: (1) sampling was conducted only in the breeding season, when individuals tend to keep their breeding territories; (2) minimum distance of 300 m between point counts; (3) counting only birds using the area within the point radius, excluding those merely flying over the area; (4) not counting groups of birds that move over large distances or are difficult to count (e.g., swallows, swifts, and birds of prey).

We sampled vegetation (height) and ground-cover variables (percentage of vegetation cover, bare ground, rocks, and water) of each point in four quadrats (1 × 1 m). These quadrats were placed at different distances from the point count: 10 m to the north, 25 m to the west, 50 m to the south, and 75 m to the east. Vegetation height was measured at five points in each quadrat (at the center and at the four vertices). These data were grouped as mean values at the point-count level. The vegetation was sampled at the end of each breeding season, immediately after the end of bird counts. This research was conducted in compliance with the requirements of the Instituto Chico Mendes de Conservação da Biodiversidade (ICMBio)—license number 49728—and Secretaria do Meio Ambiente e Desenvolvimento Sustentável (SEMA)–license number 13/2015.

### Statistical analysis

Differences in bird species richness between breeding seasons were assessed through the rarefaction and extrapolation method, based on sample coverage [48]. This method allows comparisons of richness based on samples with the same coverage (completeness) rather than the same size, which would be an advantage when comparing areas or years with very different degrees of diversity [48]. The species richness was calculated for each breeding season, based on the lowest sample coverage among the four values obtained in each history of disturbance. The 95% confidence intervals were obtained with 999 iterations by bootstrap resampling. Significant differences at the 5% level are guaranteed when the confidence intervals do not overlap [48]. This analysis was performed with the *iNEXT* package, using the *estimateD* function [49, 50].

We used generalized linear mixed models (GLMM) with a Negative Binomial error distribution to test for differences in bird abundance between breeding seasons. For vegetation height models we also used GLMM, but in this case with a Gamma error distribution and a log link function. We selected the probability distribution that best fitted the data using the *fitdist* function of the *fitdistrplus* package [51]. For the bird abundance analysis, we used only the maximum number of individuals recorded in the two samples taken in each point count per breeding season, to avoid overestimates caused by re-counting the same individual. We created models for each history of fire disturbance (occasional fire, annual fire, and without fire) separately, to assess only the temporal changes within each history, not among them. In all models the year was considered as a fixed effect, while areas and point counts were treated as random effects, to control spatial and temporal variations, considering the dependence in our data. The model analyses were carried out in the *lme4* package using the *glmer* and *glmer*.*nb* functions [52]. The significance of the fixed effect (year) was assessed via likelihood-ratio tests, with an ANOVA between the model with the explanatory variable (fixed effect) and the model without this variable (null model) [52, 53]. Differences among the four breeding seasons were evaluated via post-hoc Tukey pairwise comparisons, using the *multcomp* package [54].

In order to determine the responses of species to the time since fire (i.e. years since the last burn), we initially estimated the density (individuals/ha) of some species in occasional-fire areas, because only these areas had changes in their history of disturbance during our study. We used the Distance 7.3 program to adjust detectability issues [55]. Only species with more than 30 records were included in this analysis [56]. The estimates of density generated were tested for normality via a Shapiro-Wilk test, with the *RVAideMemoire* package [57], showing normal distribution. Therefore, we used linear mixed models (LMM) to assess the effect of the time since fire on the density of each species. In all models the year was considered as a fixed effect and the areas as random effect, accounting for temporal dependence in the data (same areas sampled in four consecutive breeding seasons/years). The model analyses were carried out in the *lme4* package, using the *lmer* function [52]. Again, the significance of the fixed effect was evaluated using an ANOVA between the model with the explanatory variable and the null model (without this variable), and differences among breeding seasons were evaluated via post-hoc Tukey pairwise comparisons.

In order to test for differences in the composition of the bird community between breeding seasons, for each history of fire disturbance, we used permutational multivariate analysis of variance (PERMANOVA) with 999 iterations, using the *Adonis* function in the *vegan* package [58]. We used post-hoc Tukey pairwise comparisons to assess differences between seasons, using the *pairwiseAdonis* package [59]. We plotted only the results for occasional-fire areas to show associations between bird species and time since fire, through a non-metric multidimensional scaling (NMDS), using the Bray-Curtis index as a dissimilarity measure, with the *metaMDS* function in the *vegan* package. Environmental variables were then fitted in the NMDS plot and their statistical significance tested with 999 permutations, using the *envfit* function in *vegan*. For this analysis, we used only grassland dependent bird species (*sensu* [1]), because there were many non-grassland dependent species with one single individual recorded (see S2 Table for a list of these species). In addition, grassland dependent species are those that would be most susceptible to changes in the grasslands, since they are dependent on this habitat. All analyses were performed using R 3.4.0 [60].

## Results

### Bird richness and abundance

We recorded 73 bird species and 2,828 individuals during the study. Of this total, 59 species and 1,325 individuals were recorded in areas with occasional fire (OF), 58 species and 1,093 individuals in areas burned annually (AF), and 34 species and 410 individuals in areas without fire (WF). Eight species were exclusive to OF, nine to AF, and four to WF. Seven other species occurred in areas with all histories of fire disturbance and in all breeding seasons. The Ochre-breasted Pipit (*Anthus nattereri*) and the Saffron-cowled Blackbird (*Xanthopsar flavus*), two species of conservation concern, did not occur in areas without fire or in the breeding season before the burn (in the case of OF), while in areas burned annually, these species were recorded in all four breeding seasons sampled. See S2 Table for a list of all bird species and their relative abundances in each year and history of fire disturbance. In addition, see S3 Table for bird abundance in each point count, site, and year.

**Fig 2:**
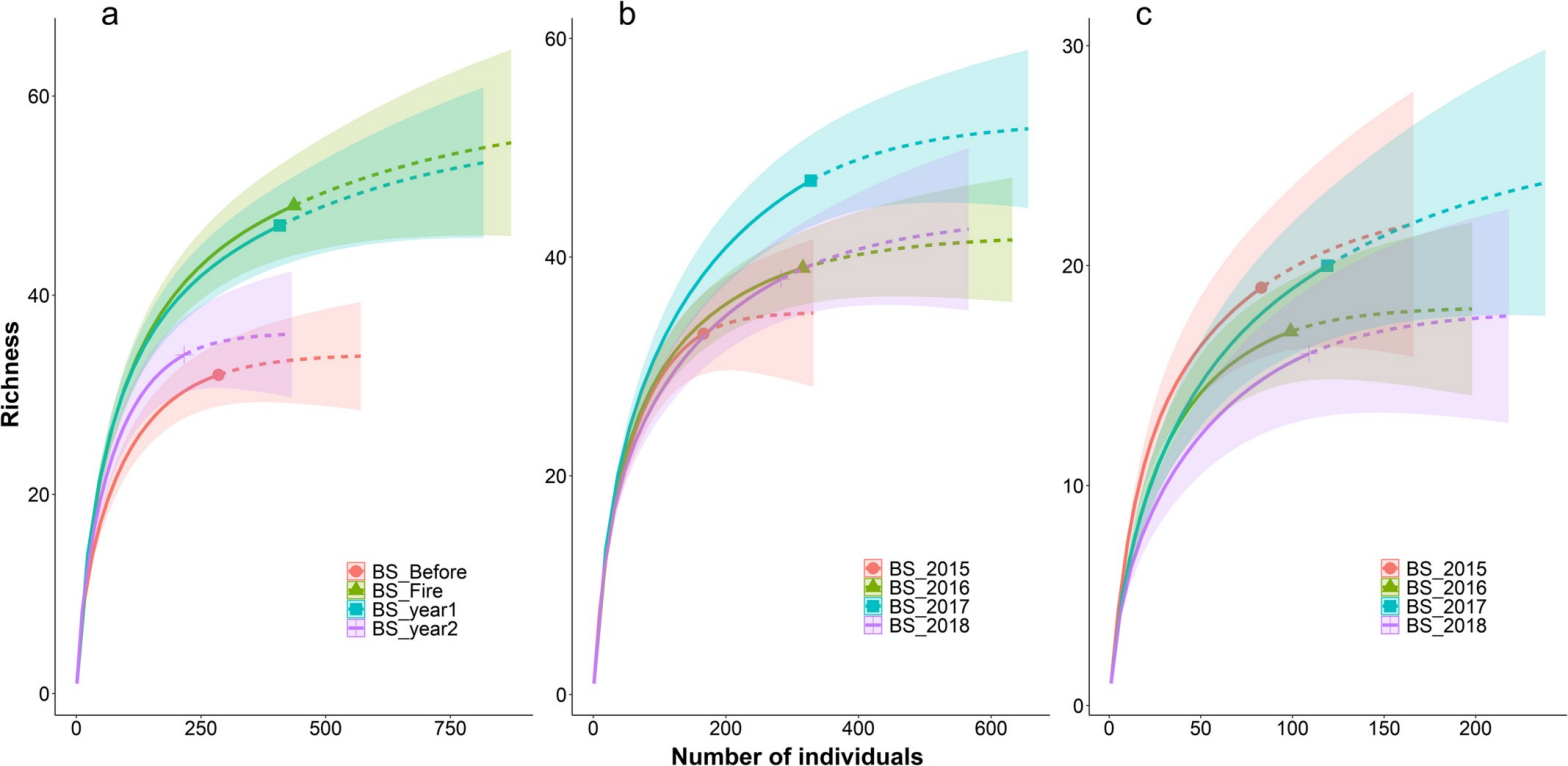
Species richness of birds recorded during four breeding seasons (BS) in grasslands with three histories of fire disturbance in northeastern Rio Grande do Sul state, Brazil. Histories of fire disturbance are occasional fire (a), annual fire (b), and without fire (c). In occasional fire breeding seasons refer to the year before the burn (before), in the year of the burn (fire), and one and two years post-fire (year1 and year2, respectively). Solid and dashed lines are interpolated and extrapolated data, respectively, based on rarefaction and extrapolation method, with their associated 95% confidence intervals.

The high values of estimated sample coverage (ranges from 0.94 in WF to 0.98 in OF and AF) indicate that the sampling was sufficient to detect most species. Considering the lowest sample coverage of each history of fire disturbance no overlap in confidence intervals showed a significant difference in richness over the breeding seasons in OF and AF (Table 1 and Fig 2). In the OF areas, richness was higher in the year of the fire (47 species) and one year post-fire (46 species), compared to the year before the burn (31 species) and two years after the burn (34 species). In the AF areas, richness was higher in the third year (2017: 47 species) than in the others (2015: 33 species; 2016: 36 species; 2018: 37 species). In the WF areas, bird richness did not vary significantly during the four years.

**Table 1:** Bird richness in areas with different histories of fire disturbance during four breeding seasons (2015–2018) in highland grasslands in northeastern Rio Grande do Sul state, Brazil.

History / Breeding season	S.obs	SC	S	CI
Occasional fire / before	32	0.98	31	27.9–33.8 ^A^
Occasional fire / fire	49	0.98	47	43.0–51.9 ^a^
Occasional fire / 1 year post-fire	47	0.98	46	41.4–50.4 ^a^
Occasional fire / 2 years post-fire	34	0.97	34	30.6–37.4 ^A^
Annual fire / 2015	33	0.96	33	29.6–36.4 ^B^
Annual fire / 2016	39	0.98	36	33.6–39.3 ^B^
Annual fire / 2017	47	0.97	47	42.3–51.0 ^b^
Annual fire / 2018	38	0.97	37	33.4–41.2 ^B^
Without fire / 2015	19	0.94	19	15.8–22.2 ^C^
Without fire / 2016	17	0.97	16	13.5–18.0 ^C^
Without fire / 2017	20	0.95	19	15.9–22.2 ^C^
Without fire / 2018	16	0.96	15	12.3–16.9 ^C^

S.obs = observed richness; SC = sample coverage; S = richness based on the lowest sample coverage for that history of disturbance; CI = 95% confidence interval. Differences between letters (upper and lower case) next to CI of a same history indicate significant differences between breeding seasons.

**Fig 3:**
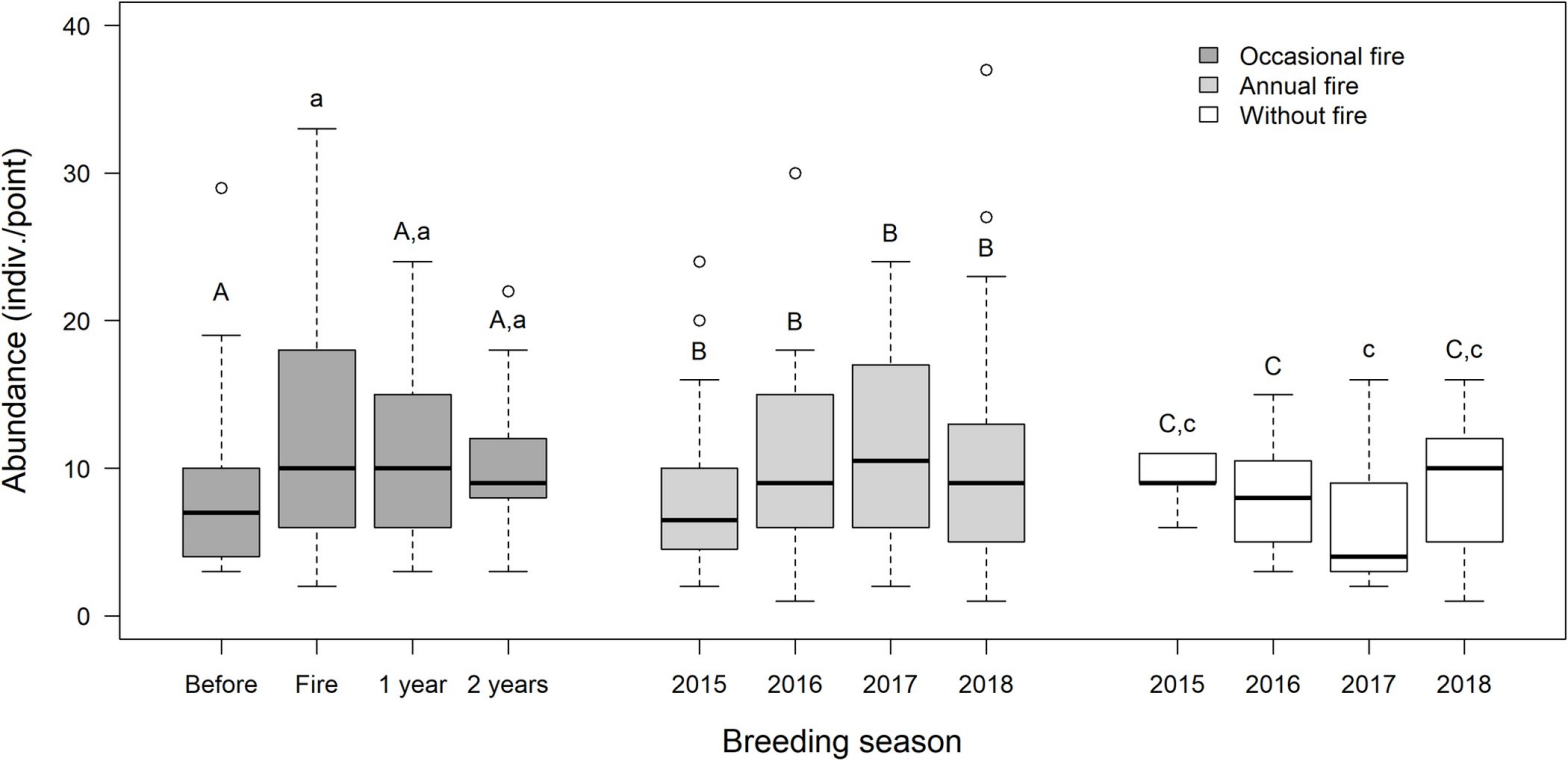
Abundance of birds recorded during four breeding seasons (2015–2018) in grasslands with three histories of fire disturbance in northeastern Rio Grande do Sul state, Brazil. In occasional fire breeding seasons refer to the year before the burn (before), in the year of the burn (fire), and one and two years post-fire (1 year and 2 years, respectively). Differences between letters in a same fire history (upper and lower case) indicate significant differences between breeding seasons, based on generalized linear mixed models. The line inside each box represents the median; top and bottom of each box represent upper and lower quartiles, respectively; whiskers represent maximum and minimum values; circles are outliers.

There was significant temporal variation in bird abundance in OF (χ^2^ = 9.11; p *=* 0.02). Post-hoc tests showed that the number of records increased in the year of the fire (Z = -3.06; p *=* 0.01), and did not differ in the following years (1 year post-fire: Z = -2.38; p *=* 0.08; 2 years post-fire: *Z* = -1.34; p *=* 0.53) in relation to the year before the disturbance (Fig 3). Bird abundance also changed significantly in WF (χ^2^ = 10.93; p *=* 0.01), where the number of records differed significantly only between the second and third years (Z = -2.61; p *=* 0.04). In AF, bird abundance did not vary significantly during the four years (χ^2^ = 3.20; p *=* 0.36).

**Fig 4:**
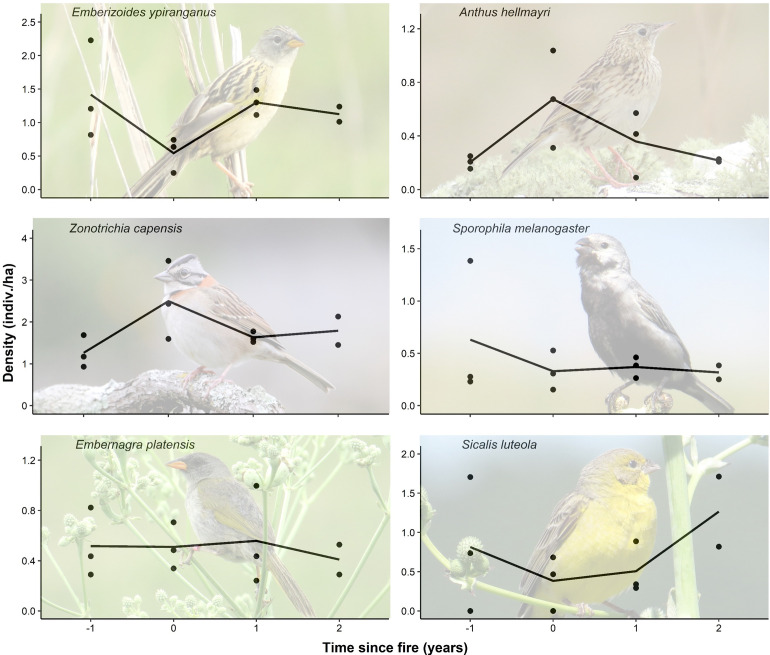
Density of six grassland birds one year before (“-1”), just following (“0”), and for two years after fire in highland grasslands in northeastern Rio Grande do Sul state, Brazil. Points represent the density of the species in the areas burned only once during the study at different times since fire. Line represents the mean density of the species in each time since fire. Only densities of *E*. *ypiranganus*, *A*. *hellmayri*, and *Z*. *capensis* varied significantly over time (p < 0.05), based on linear mixed models.

Considering the six species analyzed for density (for which the minimum number of records was obtained), three of them responded significantly in relation to time since fire (Fig 4). The time since fire positively affected the density of the Lesser Grass-Finch (*Emberizoides ypiranganus*; χ^2^ = 8.03; p *=* 0.04). Post-hoc tests showed that the density of the species was significantly higher one year post-fire (Z = 2.89; p *=* 0.02) and before the burn (Z = 3.35; p *=* 0.004) compared to the year of the disturbance. The density of the Lesser Grass-Finch did not vary between the year before the burn and one year (Z = 0.45; p *=* 0.97) or two years post-fire (Z = 0.81; p *=* 0.85). For Hellmayr's Pipit (*Anthus hellmayri*), the time since fire negatively affected its density (χ^2^ = 8.04; p *=* 0.04). The density of the species was significatively higher in the year of the fire than in the year before the disturbance (Z = -3.24; p *=* 0.007) and two years post-fire (Z = -2.74; p *=* 0.03). The density of the Hellmayr's Pipit did not differ between the year before the burn and one year (Z = -1.02; p *=* 0.71) or two years post-fire (Z = -0.14; p *=* 0.99). Similarly, the time since fire negatively affected the density of the Rufous-collared Sparrow (*Zonotrichia capensis*; χ^2^ = 7.88; p *=* 0.04). The density of the Rufous-collared Sparrow was significatively higher in the year of the fire than in the year before the disturbance (Z = -3.3; p *=* 0.005), while there was no difference between the year before the burn and one year (Z = -0.98; p *=* 0.76) or two years post-fire (Z = -1.26; *=* 0.58). For the other three species, the time since fire had no effect on their densities (*S*. *melanogaster*: χ^2^ = 1.98; p *=* 0.58; *E*. *platensis*: χ^2^ = 0.53; p *=* 0.91; *S*. *luteola*: χ^2^ = 4.23; p *=* 0.24). See S4 Table for density of the species in each site and breeding season.

### Species composition and environmental variables

The species composition of grassland birds changed over time in OF (F = 3.99, df = 3 and 121; r^2^ = 0.09; p = 0.001). Post-hoc tests revealed significant differences between the year of the burn and the other three breeding seasons (before: r^2^ = 0.11; p_adj_ = 0.006; one year post-fire: r^2^ = 0.05; p_adj_ = 0.03; two years post-fire: r^2^ = 0.10; p_adj_ = 0.006). The species composition one and two years after the burn did not differ from the year before the burn (r^2^ = 0.03; p_adj_ = 0.31; r^2^ = 0.04; p_adj_ = 0.37, respectively). Temporal changes also occurred in the species composition in AF (F = 1.73, df = 3 and 93; r^2^ = 0.05; p *=* 0.02) and in WF (F = 2.93, df = 3 and 46; r^2^ = 0.16; p *=* 0.002). In AF areas, the species composition in the first year was significantly different from the third (r^2^ = 0.06; p_adj_ = 0.02) and fourth year (r^2^ = 0.07; p_adj_ = 0.01). In the WF areas, there were significant differences between the first and third years (r^2^ = 0.13; p_adj_ = 0.04) and between the third and fourth years (r^2^ = 0.12; p_adj_ = 0.02).

**Fig 5:**
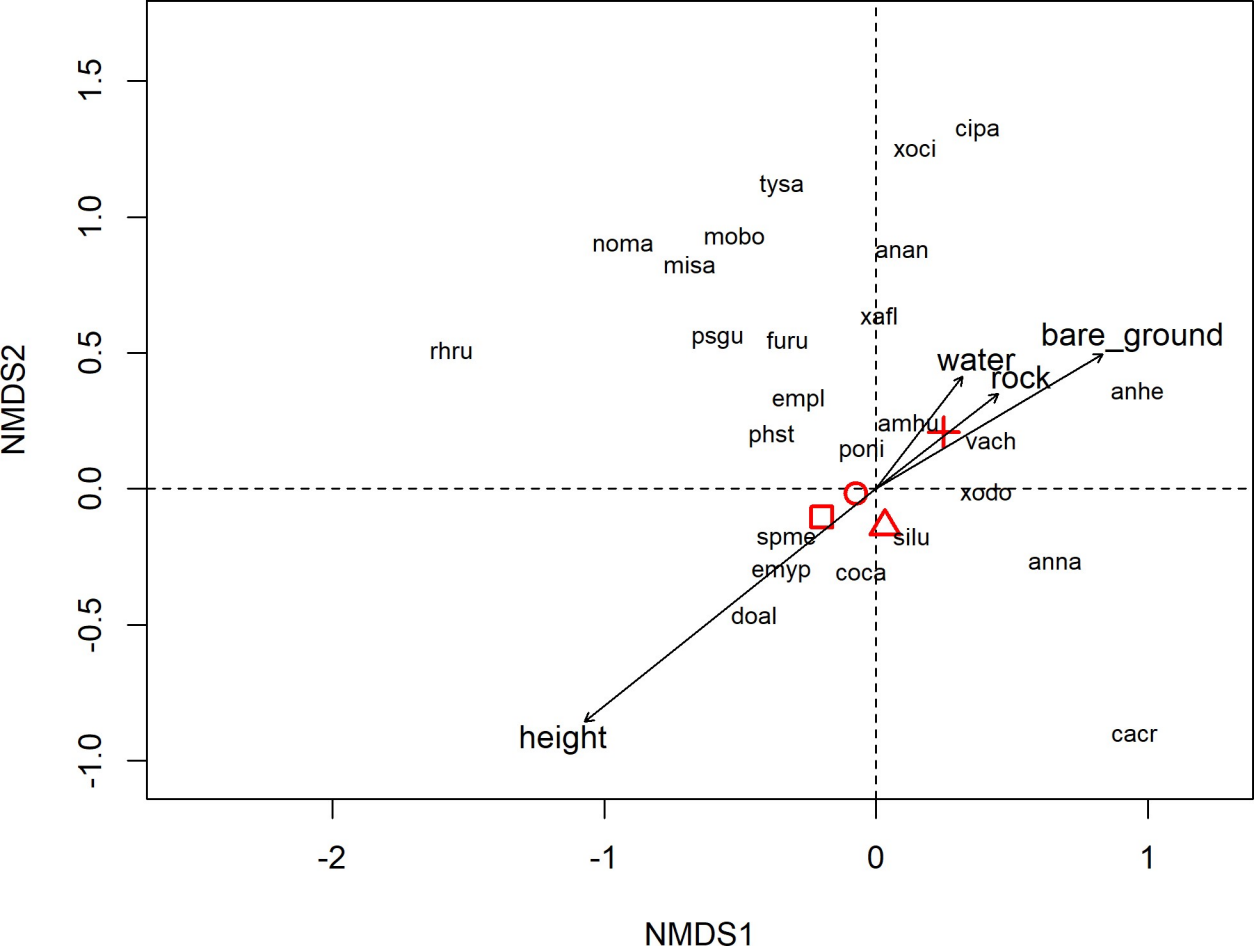
Non-metric multidimensional scaling (NMDS) plot illustrating the association among the grassland birds, four environmental variables (vegetation height, bare ground, rocks, and water) and a time gradient in relation to the fire disturbance. Results based on the species abundance (Bray-Curtis dissimilarity index) in areas with occasional fire (i.e. burned only once during the study). Stress = 0.22. Symbols represent the mean scores (centroids) of each breeding season (square = before the fire; cross = year of fire; circle = one year post-fire; triangle = two years post-fire). Acronyms are formed by the first two letters of the genus and species epithet of species in S2 Table.

As shown by the average of the breeding season scores (centroids), and reflecting the results of the tests in OF, the post-fire years (1 and 2 years) were more closely related to the year before the burn than to the year of the fire (Fig 5). Vegetation height (r^2^ = 0.24; p *=* 0.001) and percentage of bare ground (r^2^ = 0.11; p *=* 0.005) were the environmental variables significantly associated with the structure of the bird community in this history of fire disturbance. Some tall-grass species were more associated with years without burn when vegetation is taller (e.g., *Emberizoides ypiranganus*, *Sporophila melanogaster*, and *Donacospiza albifrons*), while short-grass species were more associated with the year the area was burned and, consequently, the grassland has shorter vegetation and more bare ground (e.g., *Vanellus chilensis*, *Ammodramus humeralis*, and *Anthus hellmayri*). In AF areas, vegetation height (r^2^ = 0.24; p *=* 0.001) and percentage of bare ground (r^2^ = 0.13; p *=* 0.002) also were the environmental variables associated with the species composition, while in WF areas only vegetation height (r^2^ = 0.13; p *=* 0.03) was significative. The other variables did not show significant values in none of the histories of fire disturbance evaluated. The percentage of plant cover was strongly correlated with vegetation height and was not evaluated in this analysis. See S5 Table for values of bird abundance and environmental variables in each point count, site, and year.

The vegetation height varied significantly over time in OF (χ^2^ = 135.88; p < 0.001), decreasing after the burn and increasing in subsequent years (Table 2). Post-hoc comparisons indicated significant differences among all breeding seasons (in all combinations, *Z* > 46.19; p < 0.001). There were also changes in vegetation height between the breeding seasons in AF (χ^2^ = 28.09; p < 0.001) and WF (χ^2^ = 21.63; p *=* 0.001). In AF areas, the vegetation height in the first year was significantly higher than in the second (Z = -2.87; p < 0.02) and third years (Z = -5.01; p < 0.001), and there were also differences between the third and fourth years (Z = 4.83; p < 0.001). In WF areas, the vegetation height in the first and fourth years was higher than in the second (Z = -3.91; p < 0.001; Z = 3.23; p = 0.006) and third years (Z = -4.65; p < 0.001; Z = 3.87; p < 0.001). See S5 Table for vegetation height in each point count, site, and year.

**Table 2:** Measurements of four environmental variables in areas with different histories of fire disturbance during four breeding seasons (2015–2018) in highland grasslands in northeastern Rio Grande do Sul state, Brazil.

History	Season	Vegetation height (cm)	Bare ground^a^	Rock^a^	Water^a^
Occasional fire	Before	63.2 ± 22.4 (31.2–109.6)	0.08 ± 0.45 (0–2.5)	2.04 ± 5.22 (0–25)	2 ± 6.34 (0–25)
	Fire	22.7 ± 12.2 (9.7–66.4)	15.6 ± 12.6 (1.5–47.5)	0.92 ± 2.93 (0–16.2)	1.57 ± 4.24 (0–21.2)
	1 year	34.4 ± 10.8 (15.3–54.2)	1.61 ± 3.43 (0–20.5)	0.67 ± 2.88 (0–17.5)	0.81 ± 3.50 (0–21.2)
	2 years	40.5 ± 11.2 (25.2–70.7)	0.2 ± 0.48 (0–2)	0.53 ± 1.98 (0–9.25)	0.29 ± 0.99 (0–4.5)
Annual fire	2015	25.5 ± 11.4 (7.1–46.2)	0 ± 0 (0–0)	7.51 ± 12.0 (0–40.6)	3.79 ± 8.36 (0–31.2)
	2016	20.9 ± 14.9 (9.5–75.1)	3.5 ± 3.51 (0.25–14.7)	2.74 ± 4.39 (0–20.5)	0.98 ± 3.09 (0–13.7)
	2017	16.2 ± 10.3 (5.6–38.7)	2.57 ± 3.04 (0–12.5)	3.68 ± 4.29 (0–16)	0.47 ± 0.90 (0–3.75)
	2018	24.4 ± 9.17 (9.1–40.8)	0.64 ± 1.06 (0–3.75)	2.72 ± 4.04 (0–16)	0.30 ± 0.89 (0–3.75)
Without fire	2015	59.6 ± 12.4 (41–78.8)	0 ± 0 (0–0)	1.38 ± 2.40 (0–6.25)	4.51 ± 8.43 (0–25)
	2016	46.6 ± 11.2 (26.1–61.9)	0 ± 0 (0–0)	1.14 ± 2.79 (0–8.75)	0 ± 0 (0–0)
	2017	44.7 ± 10.8 (30.8–67.8)	0.2 ± 0.41 (0–1.75)	0.14 ± 0.37 (0–1.25)	0.73 ± 1.55 (0–6.25)
	2018	50.3 ± 8.6 (31–67.5)	0 ± 0 (0–0)	0.07 ± 0.26 (0–1)	1.25 ± 4.01 (0–15)

Values correspond to mean ± SD (minimum–maximum).

^a^ Mean percentage of cover in quadrats of 1 m^2^ within bird point counts.

## Discussion

We found that the richness, abundance, and species composition in the study areas changed over time in different ways. A more regular pattern of variation was found in areas that burned occasionally, where all parameters changed in the year of the fire and returned to the same levels as in the year before the fire one or two years after the disturbance. In areas burned annually or in areas without fire, changes did not occur (e.g., richness and bird abundance did not vary in areas without fire and in areas with annual fire, respectively) or occurred in different years, without a definite temporal pattern. Lindenmayer et al. [61] also reported a different pattern of temporal changes between burned and unburned sites, where the rate of increase of bird richness was higher in burned sites.

The increase in bird richness observed shortly after the fire in areas that have gone through a long period without a burn may be related to a greater habitat heterogeneity. After a fire, the dry grassland has the shortest vegetation, while wetlands, depending on the fire intensity, are less impacted and have a different vegetation structure. The lower intensity of fire in wetlands than in dry grasslands observed in Brazilian *Cerrado* grasslands can be attributed to the higher soil water availability [62]. The heterogeneity of the vegetation structure increases the habitat variability and the diversity of the grassland bird community [16, 63]. Thus, in our study areas, species associated with tall grasses (e.g., *Emberizoides ypiranganus*, *Sporophila melanogaster*, and *Phacellodomus striaticollis*, *sensu* [1]) did not disappear after the fire, but tended to occupy areas with tall vegetation such as *Eryngium* marshes, and were often restricted to these habitats. The dry grassland, in turn, provided habitat for species associated with low grasses (e.g., *Vanellus chilensis*, *Cinclodes pabsti*, *Anthus* spp.) that did not previously occur there or were less abundant before the fire. In fact, our results showed that some species are more associated with sites with taller vegetation, while other species occupy sites with shorter vegetation and more bare ground (Fig 5).

On the other hand, in the breeding season that the areas were unburned for long periods, we observed lower bird richness compared to years when these areas had been burned (Table 1). Probably in these cases, the greater homogenization of the habitat structure, due mainly to the presence of tall grasses, disadvantages species that occupy low grasslands. Sites without disturbances for long periods usually present an increase in vegetation height and plant biomass, which leads to a homogenization of vegetation structure [15, 18]. The diversity of vegetation and structural differences, such as vegetation height, are important variables that determine the response of grassland birds [16, 32, 42, 64]. We did not record some species in areas unburned for a long period, such as the Saffron-cowled Blackbird (*X*. *flavus*) and the Ochre-breasted Pipit (*A*. *nattereri*), two globally threatened species [65]. In the Brazilian Cerrado, *A*. *nattereri* has been recorded in native grasslands affected by fire [66, 67]. Grasslands with more than two years of post-fire succession and no grazing, even in sites with favorable relief, do not seem to favor the occurrence of the Ochre-breasted Pipit [68]. In southern Brazil, the Saffron-cowled Blackbird used burned areas more frequently and avoided habitats with tall grasses and developed vegetation [37]. The species was absent from a protected area that has not experienced fires in nearly three decades [37], since the Saffron-cowled Blackbird depends on marshes to breed but also uses dry, short-grass areas to forage [37, 69, 70]. In Argentina, Isacch and Martinez [30] observed that areas with more tall-grass coverage had higher richness and abundance of birds. However, the authors noted that they did not sample sites with 100% tall-grass coverage (probably equivalent to WF areas in our study), and that in this situation, the richness is likely lower due to the loss of ground-feeding species.

In grasslands of the Serra da Canastra National Park, southeastern Brazil, burnings triggered profound and immediate changes in bird assemblages, increasing the number of species and individuals right after the fire [35]. Bahía and Zalba [32] found that the abundance and richness of birds were lower one year after a burn and increased significantly two years afterward. Besides the increase in richness, our data also showed an increase in the abundance of individuals after a fire. Probably the occurrence of some common species in our areas, such as the Rufous-collared Sparrow (*Z*. *capensis*), Hellmayr's Pipit (*A*. *hellmayri*), and Southern Lapwing (*V*. *chilensis*) contributes to this higher abundance. In North America, some species occurred in higher abundances in areas with fire disturbance [16, 24, 25].

Three species assessed individually showed responses to fire, in different ways. The densities of Rufous-collared Sparrow and the Hellmayr's Pipit (a non-grassland and a short-grass species, respectively, according to [1]) increased with a burn and later decreased over time after the fire. In contrast, the density of the Lesser Grass-Finch (a tall-grass species) decreased in the year of the fire, increasing again as the time since the fire lengthened (Fig 4). This is expected and occurs because some species are favored and others are disadvantaged by fire disturbance, responding to variations in habitat structure [28, 30]. Lesser Grass-Finch tends to be disadvantaged by fire disturbance due to the loss of suitable habitat, since it depends on a specific vegetation structure to nest, forage, and seek refuge [71]. Some species of conservation concern that occur in the region of our study might be more frequent and abundant in areas burned frequently (e.g., *Cinclodes pabsti*, *Anthus nattereri* and *Xanthopsar flavus*), where short grasses are predominant, while others might be more frequent and abundant in areas with reduced fire management or without it (e.g., *Scytalopus iraiensis*, *Limnoctites rectirostris* and *Sporophila melanogaster*), where vegetation is higher [39]. Although we assumed that grazing pressure was constant at our study sites, it is important to note that grazing influences directly the vegetation structure and plant taxonomic diversity, promoting effects on plant and arthropod communities [15], and, consequently, may have additional effects on grassland birds. Several tallgrass-dependent birds are threatened in South America and are affected by the lack of tallgrass vegetation caused by the intensive disturbance of grassland due to cattle raising [1, 42].

The present results also showed changes in grassland-bird species composition over the years, but the spatial dependence of the data used in the analysis requires caution in interpreting the effects of fire on community structure. Vegetation height was the main variable associated with species composition, corroborating other studies (e.g., [42, 72]). Our data point to an effect of fire in areas that burned occasionally, with a significant difference in the year of the burn compared to the others. Differences in species composition were also found among the years in areas burned annually and in areas without fire, without a definite pattern in these fire regimes. Annual variation in areas without changes in the fire regime over the years may be the result of climatic variation. Temporal fluctuations of bird communities can be indirectly caused by both changes in temperature and precipitation, which determine the amount of resources available to birds [73, 74]. Areas burned annually, for example, may have variable amounts of primary productivity, depending on rainfall and temperatures, affecting the stability of the grassland bird community [17].

Although caution is needed for extrapolations due to the small number of areas that it was possible to sample, our results converge to the ideas that grasslands should be managed in a way that forms mosaics with a spatial and temporal arrangement of both short and tall grass, creating vegetation heterogeneity and promoting bird diversity [16, 17, 42]. Patch-burn management has been recommended for grassland bird conservation, because it creates the entire gradient of vegetation structure required to maintain grassland bird species that differ in habitat preferences [16, 75]. Thus, management of grasslands that creates a shifting mosaic, using prescribed fires in areas with different times since burnings and areas with different histories of fire disturbance, can be useful in the conservation of grassland birds and habitats. Fire in highland grasslands of southern Brazil has already been recommended as an important management tool to ally cattle raising with bird conservation [39].

This study is the first to use data covering the period before and after a burn in southern Brazilian grasslands, since we are not allowed to burn large areas for experiments, especially in Brazilian protected areas. We took advantage of three events of occasional (accidental) fire in the protected areas where we usually develop bird monitoring to answer relevant questions to grassland conservation in southern Brazil. Our results showed that two years without fire was a period long enough for the evaluated parameters of the bird community and the density of some species to return to levels estimated before the disturbance. The recovery of the vegetation after a fire and the response time of the birds, in one or two breeding seasons, depending on the species, were also observed in Argentina [32]. In southeastern Australia, an endangered bird species either remained continuously on burned sites or returned to previously occupied sites within two years after an unplanned fire [76]. Another study in Argentina, with a threatened grassland bird, found that the species did not show avoidance of the burned patch in the third breeding season after the prescribed fire, suggesting burning intervals longer than two years [31]. This kind of information is useful for planning the periodicity with which fire can be used to manage grassland areas.

We showed that the use of fire in highland grasslands of southern Brazil should consider a period of at least two complete years (or at least two breeding seasons) without burnings in the same grassland patch, to ensure the recovery of the bird community of the area. Another point of concern is that fire must occur before the breeding season of most grassland birds (which is the austral spring and summer in southern Brazil [46, 77]) to avoid burning active nests. Additionally, fires must not affect wetlands to avoid losing important sites of refuge and/or nest sites for tall-grass birds, including migratory and philopatric species such as *Sporophila melanogaster*. However, we highlight the necessity of additional researches with a larger number of areas (replicates) to reinforce our findings. Moreover, management decisions and a better understanding of the effects of fire require an analysis that integrates different taxonomic groups of animals and plants.

## Supporting information

S1 Table Areas used to sample birds in highland grasslands in northeastern Rio Grande do Sul state, Brazil. Numbers in each breeding season represent the years since fire (“0” indicates the year when the area was burned) and the quantity of point counts sampled (between parentheses). Sites: APA = Aparados da Serra region, TAI = Tainhas State Park region.
(XLSX)

S2 Table Relative abundance (number of individuals/point) of bird species recorded during four breeding seasons (2015–2018) in grasslands with three histories of fire disturbance (occasional fire, annual fire, and without fire) in northeastern Rio Grande do Sul state, Brazil. In occasional fire breeding seasons refer to the year before the burn (before), in the year of the burn (fire), and one and two years after a fire (1year and 2years, respectively). Species are in alphabetical order. In bold are grassland dependent bird species.
(XLSX)

S3 Table Bird abundance across in each point count, site, and year during four breeding seasons in grasslands with three histories of fire disturbance. In occasional fire breeding seasons refer to the year before the burn (before), in the year of the burn (fire), and one and two years after a fire (1year and 2years, respectively). Sites: APA = Aparados da Serra region, TAI = Tainhas State Park region.
(XLSX)

S4 Table Density (individuals/ha) of six bird species across in each site and year during four breeding seasons in areas with occasional fire in highland grasslands of northeastern Rio Grande do Sul state, Brazil. Breeding season refer to the year before the burn (before), in the year of the burn (fire), and one and two years after a fire (1year and 2years, respectively). Sites: APA = Aparados da Serra region, TAI = Tainhas State Park region. Density calculated using the Distance 7.3 program.
(XLSX)

S5 Table Environmental variables and abundance of grassland-dependent bird species across in each point count, site, and year during four breeding seasons in grasslands with three histories of fire disturbance (occasional fire, annual fire, and without fire). In occasional fire breeding seasons refer to the year before the burn (before), in the year of the burn (fire), and one and two years after a fire (1year and 2years, respectively). Sites: APA = Aparados da Serra region, TAI = Tainhas State Park region. Acronyms of bird species are formed by the first two letters of the genus and species epithet of species in S2 Table.
(XLSX)
